# Life-Threatening Anaphylactoid Reaction Manifesting as Ventricular Arrhythmia and Pulmonary Edema Following Methylene Blue Injection During Chromopertubation: A Case Report

**DOI:** 10.7759/cureus.99823

**Published:** 2025-12-22

**Authors:** Ankita Kabi, Vijeta Bajpai, Priyanka Dwivedi, Sonam Patel

**Affiliations:** 1 Anesthesiology, All India Institute of Medical Sciences, Gorakhpur, Gorakhpur, IND

**Keywords:** anaphylactoid reaction, chromopertubation, methemoglobinemia, methylene blue dye, pulmonary edema, ventricular arrhythmia

## Abstract

Methylene blue dye is commonly used for chromopertubation during diagnostic laparoscopic procedures. Although this dye is considered safer in diluted doses, several cases of cardiovascular complications, including collapse, allergic reactions, and methemoglobinemia due to methylene blue dye, have been reported. We report a case of unstable ventricular arrhythmia followed by delayed pulmonary edema in the postoperative period with a very low dose of methylene blue. A 29-year-old female with secondary infertility underwent diagnostic hysterolaparoscopy under general anesthesia. Diluted methylene blue dye (30 mL of 0.5 mg/mL) was administered transcervically to assess tubal patency, confirming bilateral tubal blockage without dye spillage. Three minutes after dye injection, the patient suddenly developed unstable ventricular tachycardia with cardiovascular compromise (blood pressure, 72/40 mmHg) and required vasopressor support for stabilization. The patient was extubated uneventfully and shifted to the postoperative ward on vasopressor support. After one hour, the patient developed tachypnea with increased oxygen requirement. Acute pulmonary edema was diagnosed based on findings of bilateral coarse crepitations, more than six B-lines on point-of-care ultrasound, and normal cardiac contractility. The patient was managed with noninvasive positive-pressure ventilation via mask, along with close monitoring of vital signs and urine output. Her respiratory and cardiovascular status stabilized within five hours. This rare combination of intraoperative ventricular arrhythmia and delayed pulmonary edema after low-dose methylene blue highlights the need for heightened vigilance and readiness for prompt resuscitative support during chromopertubation, even with very small doses of methylene blue.

## Introduction

Methylene blue is widely employed for detecting tubal patency, fistulas, and sentinel lymph node biopsies because of its tissue-staining capabilities [1]. In laparoscopic chromopertubation, methylene blue dye is introduced through the cervix to assess tubal patency [2].

Although this dye is considered safe in chromopertubation, complications such as fatal anaphylactic or anaphylactoid reactions [3-5], pulmonary edema [6], and methemoglobinemia [7,8] have been reported. The incidence of allergic reactions to blue dyes ranges from 0.07% to 2.7% in sentinel lymph node biopsy [4], but the incidence of these reactions due to methylene blue in chromopertubation is not clearly known at present. While larger doses of methylene blue can induce methemoglobinemia and potentially cardiac arrhythmias, the development of ventricular tachycardia (VT) with a very low dose of methylene blue intraoperatively during chromopertubation, along with delayed pulmonary edema, has not been reported to date.

Here, we present a case in which a patient developed unstable VT and delayed pulmonary edema following a very low dose of methylene blue injection.

## Case presentation

A 29-year-old ASA I female (BMI 21.2 kg/m²) presented with secondary infertility for nine years, with one prior successful pregnancy. The patient was scheduled for hysterolaparoscopy with chromopertubation under general anesthesia to identify the cause of secondary infertility. She had no past history of chronic illness or allergy. Preoperative assessment was normal, and the patient was not on any medications.

General and physical examinations before the procedure showed no abnormalities. The results of preoperative investigations, including complete blood counts, serum electrolytes, electrocardiograms, and chest radiography, were within normal limits.

Prior to induction, she received IV glycopyrrolate (0.2 mg) and midazolam (2 mg) as premedication. Anesthesia induction comprised injection of propofol (2 mg/kg) and fentanyl (100 mcg), followed by intubation with a cuffed endotracheal tube (size 7.0) after administration of vecuronium (6 mg). Intraoperatively, anesthesia was maintained with a mixture of O₂:air (50:50), 2% sevoflurane, and vecuronium (0.01 mg/kg). Boluses of IV fentanyl (25 mcg) were administered for analgesia. On laparoscopy, flimsy uterine adhesions and beaded fallopian tubes were observed. Until this time, the patient was hemodynamically stable, maintaining normocapnia, adequate intravascular volume, and an adequate depth of anesthesia.

To assess tubal patency, 5 mL of 1% methylene blue (10 mg/mL) was diluted in 100 mL of saline (0.5 mg/mL), and a total of 30 mL (≈15 mg methylene blue) was injected intrauterine with resistance over three minutes. No dye spillage was observed, suggesting bilateral tubal occlusion. Hemodynamics were stable until dye injection, and no other drug was coadministered prior to the event.

Immediately afterward, the patient developed VT with an irregular pulse and hypotension. For immediate stabilization, a 250 mL saline bolus, 100% O₂, a 50 µg bolus of phenylephrine, 125 mg of IV hydrocortisone in view of a suspected anaphylactic reaction, and a norepinephrine infusion (0.06 µg/kg/min) were administered, with simultaneous preparation for cardioversion after a diagnosis of unstable VT was made. Phenylephrine was preferred over epinephrine because hypotension with tachyarrhythmia was present, and epinephrine could have worsened the condition. Vital signs stabilized, and the arrhythmia spontaneously reverted to sinus rhythm before cardioversion was administered.

**Figure 1:**
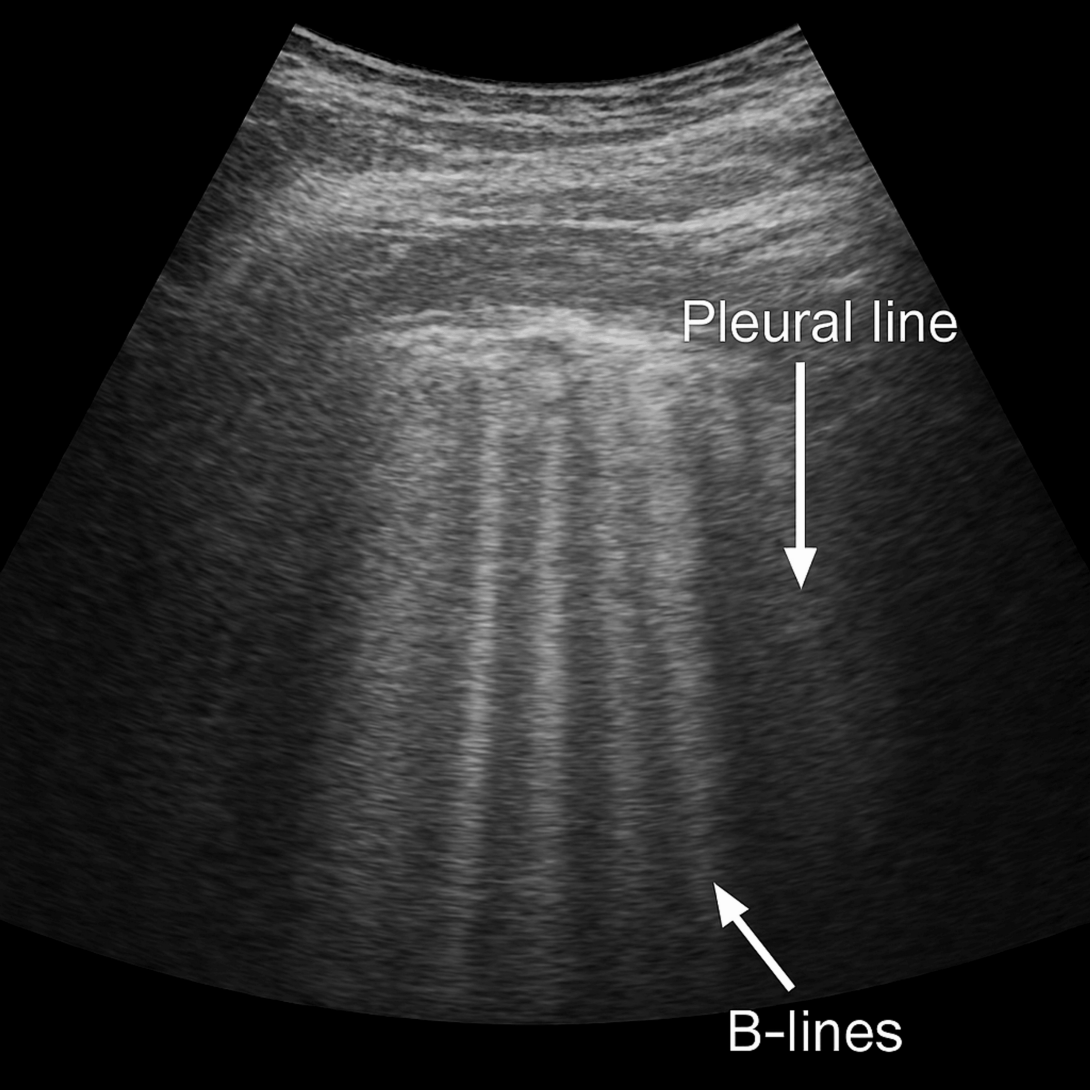
Lung ultrasonography demonstrating more than three B-lines (comet-tail artifacts) in a single intercostal space, indicative of pulmonary edema

Postoperatively, the patient remained stable with minimal vasopressor support (norepinephrine infusion, 0.06 µg/kg/min) and maintenance Ringer’s lactate infusion titrated according to hemodynamic parameters and hourly urine output. After one hour, she developed tachypnea (respiratory rate, 24/min; blood pressure, 96/60 mmHg) with an increased O₂ requirement to maintain SpO₂ at 95%. Bilateral coarse crepitations were present. Point-of-care ultrasound demonstrated more than six B-lines (Figure 1) bilaterally, consistent with acute pulmonary edema. The subcostal view demonstrated preserved cardiac function and contractility, partially excluding a cardiac etiology. A low dose of furosemide (10 mg IV) was administered initially, to be escalated as needed, and noninvasive positive-pressure ventilation via mask (FiO₂/inspiratory positive airway pressure/expiratory positive airway pressure = 0.4/12/5) was initiated. With supportive management, oxygenation and hemodynamics improved, and the patient was gradually weaned off ventilatory and vasopressor support within five hours.

With the possibility of dye extravasation beyond gentle resistance and systemic absorption causing cardiovascular collapse, the differential diagnoses included allergic or anaphylactoid reaction, methylene blue-induced cardiotoxicity, and methemoglobinemia.

Methylene blue cardiotoxicity is rare and usually occurs with large systemic doses. As only 15 mg was used, far below the toxic threshold of 7 mg/kg, this cause was ruled out. The absence of bluish discoloration of the skin or body fluids and a methemoglobin level of <0.1% on arterial blood gas analysis excluded methemoglobinemia.

Because hemodynamic instability occurred within three minutes of dye instillation, with no other drug coadministered at that time, an allergic or anaphylactoid reaction to the dye was considered the most likely cause of cardiovascular compromise. Although there was an absence of cutaneous manifestations, it is well known that skin signs may be absent under general anesthesia.

Among the possible causes of delayed pulmonary edema, fluid overload was considered; however, under strict vital sign monitoring guided by hourly urine output with a target of 0.5-1 mL/kg/hour, this was less likely to have caused pulmonary edema. Intrapulmonary vascular spasm and capillary leak secondary to an anaphylactoid reaction to methylene blue [9,10] were probable mechanisms, as the dye can induce pulmonary vasospasm and generalized vasoconstriction. The patient was managed for an allergic/anaphylactoid reaction with IV fluids, steroids, vasopressors, and ventilatory support and responded well.

The patient was monitored in the ICU for 24 hours and then shifted to the ward. She maintained stable vital signs and was discharged in good condition, with advice for follow-up in the obstetrics outpatient department for infertility management.

## Discussion

Tubal blockage or dysfunction is a primary contributor to both primary and secondary infertility, affecting about 30% of cases [1,2]. Methylene blue, because of its comparable accuracy, easy availability, lower cost, and relative safety within therapeutic doses (<2 mg/kg), is the most commonly used dye. However, it has been reported to cause both minor clinical effects, such as bluish papules and urine discoloration [5], as well as major adverse events, including sudden collapse or fatality during chromopertubation [3-6]. At high doses (>7 mg/kg), it can induce toxicity, including cardiac arrhythmias and impaired gas exchange.

Allergic hypersensitivity reactions, such as anaphylactic and anaphylactoid responses, are more commonly associated with hemodynamic instability and shock and typically occur within 10 minutes of dye injection or during the immediate postoperative period. Dewachter et al. [3] reported a severe anaphylactic reaction within two minutes of methylene blue dye instillation, associated with bronchospasm, a fall in oxygen saturation, hemodynamic instability, and generalized urticaria. Veerendrakumar et al. [9] reported a case of an anaphylactoid reaction causing pulmonary edema five hours postoperatively.

Methemoglobinemia, another reaction, typically presents with bluish discoloration of bodily fluids (stool, saliva, vomitus, and urine) and changes in pulse oximetry, with skin necrosis usually occurring after 10 minutes post-injection. It is commonly reported in patients with tuberculosis [7] or glucose-6-phosphate dehydrogenase deficiency. [8] Methemoglobin levels below 0.1% on arterial blood gas analysis, along with the absence of discoloration of tissues, urine, or skin and no changes in pulse oximetry or PaO₂, ruled out this complication.

The rapid onset of symptoms after injection suggests an allergic reaction with cardiovascular compromise. The patient required vasopressor support during and after surgery. Subsequently, the patient developed pulmonary edema one hour postoperatively, indicating a potential anaphylactoid reaction. In previously reported cases, patients developed either an allergic reaction or pulmonary edema in isolation. However, in this case, an intraoperative life-threatening reaction to methylene blue progressed to pulmonary edema in the postoperative period.

Serial serum tryptase levels could have been measured to aid in the diagnosis of an allergic reaction, as they help confirm mast cell activation and are detectable in serum within minutes of anaphylaxis or allergic reactions; however, this was not performed in the present case. This case highlights that injection against resistance may increase the risk of transtubal venous intravasation, facilitating a rapid systemic bolus, particularly in high-risk patients such as those with chronic pelvic inflammatory disease or tuberculosis. Strategies such as slow, low-pressure injection; preprocedure allergy preparedness, including having epinephrine drawn up and ready prior to dye instillation; and vigilant monitoring can help prevent and better manage such situations.

This case also underscores the importance of prolonged postoperative monitoring (e.g., four to six hours), even after apparent intraoperative stabilization, as delayed pulmonary edema can occur. For future procedures in this patient, consideration of alternative dyes (e.g., indigo carmine, though also associated with risk) or saline infusion sonography may be appropriate.

Thus, this case highlights the importance of vigilance by both the anesthesiologist and surgeon throughout the perioperative period whenever this dye is administered, along with prompt cardiovascular support if needed, especially in day-care settings with ongoing postoperative follow-up. This proactive approach can help avert morbidity and mortality associated with its use.

Treatment primarily focuses on supportive care. Steroids have shown efficacy in managing allergic reactions, while diuretics are beneficial for pulmonary edema. In cases of near-fatal anaphylactic shock, the use of extracorporeal membrane oxygenation has also been employed and shown to be lifesaving [4].

## Conclusions

The administration of methylene blue for chromopertubation, even in small, diluted doses, can precipitate immediate, life-threatening ventricular arrhythmias and delayed pulmonary edema. This case underscores that no dose is without risk. Clinicians must remain vigilant for the possibility of systemic extravasation, administer the dye slowly under low pressure, and be prepared with immediate advanced resuscitation capabilities and prolonged postoperative monitoring.
